# Incorporating lesion-to-lesion heterogeneity into early oncology decision making

**DOI:** 10.3389/fimmu.2023.1173546

**Published:** 2023-06-07

**Authors:** Rukmini Kumar, Timothy Qi, Yanguang Cao, Brian Topp

**Affiliations:** ^1^ Vantage Research Inc., Lewes, DE, United States; ^2^ Division of Pharmacotherapy and Experimental Therapeutics, Eshelman School of Pharmacy, The University of North Carolina at Chapel Hill, Chapel Hill, NC, United States; ^3^ Lineberger Comprehensive Cancer Center, The University of North Carolina at Chapel Hill, Chapel Hill, NC, United States; ^4^ Quantitative Pharmacology & Pharmacometrics, Immuno-oncology, Merck & Co., Inc., Rahway, NJ, United States

**Keywords:** QSP model, lesion-to-lesion heterogeneity, RECIST v1.1, dissociated response, oncology clinical trials

## Abstract

RECISTv1.1 (Response Evaluation Criteria In Solid Tumors) is the most commonly used response grading criteria in early oncology trials. In this perspective, we argue that RECISTv1.1 is ambiguous regarding lesion-to-lesion variation that can introduce bias in decision making. We show theoretical examples of how lesion-to-lesion variability causes bias in RECISTv1.1, leading to misclassification of patient response. Next, we review immune checkpoint inhibitor (ICI) clinical trial data and find that lesion-to-lesion heterogeneity is widespread in ICI-treated patients. We illustrate the implications of ignoring lesion-to-lesion heterogeneity in interpreting biomarker data, selecting treatments for patients with progressive disease, and go/no-go decisions in drug development. Further, we propose that Quantitative Systems Pharmacology (QSP) models can aid in developing better metrics of patient response and treatment efficacy by capturing patient responses robustly by considering lesion-to-lesion heterogeneity. Overall, we believe patient response evaluation with an appreciation of lesion-to-lesion heterogeneity can potentially improve decision-making at the early stage of oncology drug development and benefit patient care.

## Introduction

Patients with stage IV cancer generally have primary lesions as well as metastatic lesions spread across multiple organs. Mounting evidence shows that each lesion differs in genetic mutations, clonal composition, pathophysiology, and this complexity results in differential response to therapy (1–4). The present method for scoring response to therapy, RECISTv1.1, yields a patient-level response based largely on an aggregate change in the sum of target lesion diameters without an appropriate appreciation of lesion-to-lesion heterogeneity. Here, we argue that tracking aggregate change leads to bias in decision making. Therefore, we advocate for lesion-level analysis in drug development decision-making (Go/No-Go decisions, biomarker analysis, identifying combination strategies) and to potentially inform clinical drug adjustment decisions. Further we also show Quantitative Systems Pharmacology (QSP) modeling approaches that explicitly include multiple lesions can be used for decision support.

## Overview of RECIST v1.1

The Response Evaluation Criteria in Solid Tumors (RECIST) criteria are a set of guidelines for evaluating patient response to oncology treatment (5). There are several versions of RECIST, with version 1.1 being the standard method applied to virtually all oncology trials for solid tumors. Patient responses are classified into one of four strata: Complete Response (CR), Partial Response (PR), Stable Disease (SD), or Progressive Disease (PD). This classification occurs each time a patient receives a CT or MRI scan (usually every 6-8 weeks) and repeats until the end of the trial, patient death, or loss of follow-up.

The definition of each RECISTv1.1 response classification is shown in **Table 1**. When patients have multiple lesions, a subset of lesions is designated “target” lesions and measured at each scan (RECIST guidelines recommend target lesions should be representative lesions amenable to repeated measurement, up to 5 and no more than 2 per organ (5). The sum of their diameters is tracked and evaluated to determine patient response. The remaining lesions are designated “non-target” lesions and simply reported as present, absent, or progressing. If the number of lesions increases during the trial, these lesions are defined as new metastatic lesions. Complete Response requires the elimination of all target and non-target lesions and the absence of any new metastatic lesions. Partial Response requires a >30% reduction in the sum of target lesion diameters and the absence of non-target growth or appearance of new lesions. Disease progression is more complex. A patient is assigned a RECISTv1.1 classification of Progressive Disease if they show >20% growth in target lesions and/or unequivocal progression of non-target lesions and/or the appearance of new metastatic lesions.

**Table 1:** RECIST v1.1 criteria for patient classification.

	Δ Change in the Sum of diameters of Target Lesions	Non-Target Lesions	New Lesions
Complete Response^#^	-100%	Absent	Absent
Partial Response^#^	<-30%	Not Progressing	Absent
Stable Disease^#^	-30% to + 20%	No Progressing	Absent
Progression*	>+20%	Progressing	Present

^#^All three conditions are required.

*PD is declared if any one of these three conditions are met.

### Biases in RECISTv1.1

**Figure 1:**
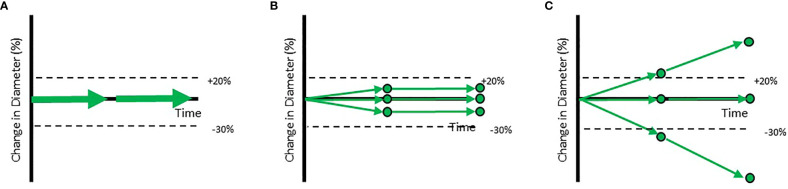
Example trajectories of patients with a RECISTv1.1 classification of Stable Disease. Each of the circles represents individual lesions in the patients. The aggregate response is represented by arrows. **(A)** Shows the standard visualization of aggregate response to therapy based on the Sum of Longest Diameters. **(B)** Shows a patient with a homogeneous response while **(C)** shows a patient with a heterogeneous response at the lesion level. When aggregate diameter changes by >20% (shown in labelled dotted line), the patient is classified as Progressive Disease. When aggregate diameter changes by < -30% (shown in labelled dotted line), the patient is classified as a Partial Responder. When all lesions have disappeared, the patient is classified as a Completer Responder.

Patient level outcomes tracked by RECIST hide important individual lesion level dynamics. Trial outcomes are commonly visualized by so-called “spaghetti plots” that display the change in the sum of lesion diameters for each patient over time (**Figure 1A**). End users may read these graphs as if they were derived from homogeneous responses across all lesions in the patient (**Figure 1B**). However, the reality is generally more complex (**Figure 1C**). While the patients in **Figures 1B, C** receive the same RECISTv1.1 classification (Stable Disease), these patients are different in clinically meaningful ways; inferences of drug efficacy and treatment approach should differ for each of them.

**Figure 2:**
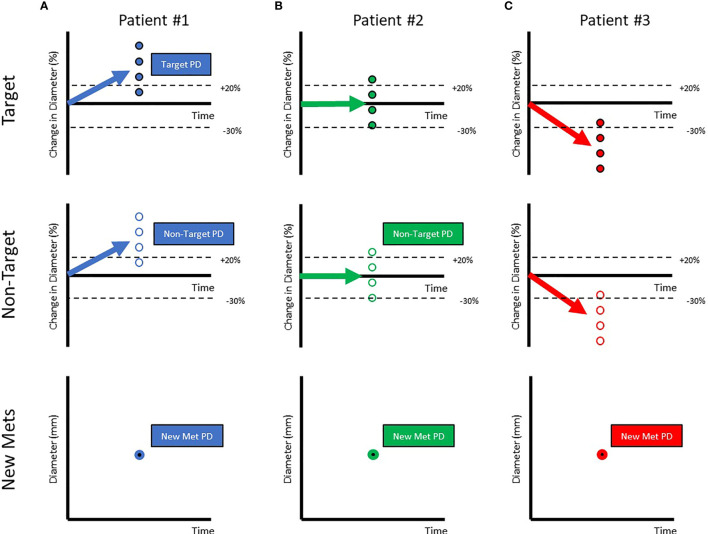
Patients classified as Progressive Disease can be very variable in the response of their target, non-target & appearance of metastatic lesions. Each of the circles represents individual lesions in the patients (Target Lesions: Filled Circles, Non-target Lesions: Open Circles, New Metastatic Lesions:Filled with bold border). The aggregate response is represented by arrows. When aggregate diameter changes by >20% **(A)** (shown in labelled dotted line), the patient is classified as Progressive Disease. When aggregate diameter changes by < -30% **(B)** (shown in labelled dotted line), the patient is classified as a Partial Responder. When all lesions have disappeared, the patient is classified as a Completer Responder **(C)**.

A RECISTv1.1 classification of Progressive Disease (PD) is generally considered drug failure (a lack or loss of efficacy). Three examples of PD are provided in **Figure 2**. The first patient is a prototypical example of drug failure. Every target lesion grows, every non-target lesion grows, and new metastatic lesions appear. Clearly, the investigational drug displays minimal or no efficacy in this patient. In the second patient, half of the target lesions grow while the other half shrink. This results in a classification of PD via non-target progression and/or the appearance of new metastatic lesions. Despite a RECISTv1.1 classification of PD, this patient gained meaningful benefit in half of their lesions. Assuming the goal is to minimize or eliminate *all* lesions, this investigational drug may be effective as part of a combination therapy. The third patient gains meaningful benefit in all pre-existing tumors but is classified as PD due to the appearance of a new metastatic lesion. New metastatic lesions can appear transiently before stabilizing or shrinking shortly after appearance (6). Although the drug displays clear and continuing efficacy in most lesions, it is considered to have failed the patient by RECISTv1.1.

Criteria such as iRECIST have been developed to account for some variability seen in response to immunotherapy. The bias with new transient lesion can be avoided by using iRECIST criteria due to the need for PD classification to be confirmed on a follow-up scan (7). However, variability among the target lesion response will still not be captured by current scoring frameworks. Additional regulatory guidance may lead to broader adoption of iRECIST & other novel scores by drug development companies, thereby leading to better decision-making.

A recent study analyzing continuing effect of ICI (anti PD1) therapy in patients classified as PD from multiple trial data concluded that treatment beyond progression with ICI might be appropriate for selected patients (8). Adding a combination therapy to address progression while maintaining the original therapy to control responding lesions is being attempted as a strategy in several recent trials (9, 10). Results from such trials will inform future directions for clinical care.

**Figure 3:**
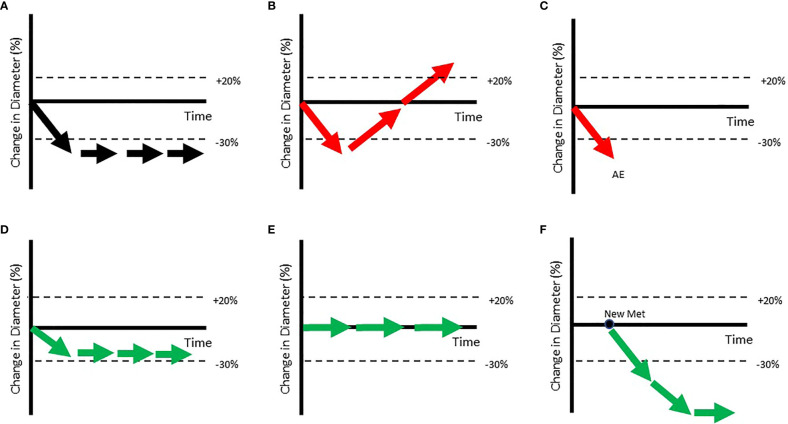
Patients with very different trajectories can be classified as Objective Responders. **(A)** Shows a prototypical Objective Responder. However, **(B, C)** show patients classified as Objective Responders who may not show such ideal trajectories (shown in red arrow). In **(B)** a patient who briefly shows reduction is classified as an Objective Responder as duration of response is not accounted for when RECIST response is assigned. In **(C)** the patient is classified as an Objective Responder, even though they dropped out at the first point due to an Adverse Event (AE). Others who arguably benefit may still not be classified as Objective Responders (shown in green arrows in bottom row). **(D)** Shows a patient whose tumor has stabilized just above the dSLD < -30% threshold. **(E)** Shows a patient who shows clear benefit from the therapy as tumor growth is inhibited but will be considered a non-responder as the lesion has not shrunk. **(F)** Shows a patient with a new metastatic lesion who will be classified as Progressive Disease even though that lesion may shrink on further treatment.

In addition to vastly different patients being classified as PD, RECISTalso describes vastly different patients as Objective Responders (Complete or Partial Response). **Figure 3A** shows a prototypical Objective Responder. This patient is expected to gain a survival benefit from the investigational drug. However, **Figure 3B** shows an Objective Responder whose disease rebounds early in the trial and is unlikely to experience meaningful benefit. Since RECIST captures the “best overall response”, this patient can be classified as a responder. **Figure 3C** shows another Objective Responder that drops out of the trial early due to an adverse event. Again, this patient does not display an ideal response to therapy but is classified as Objective Responder. **Figure 3D** shows a patient that gains a stable 29% reduction in tumor burden but is *not* classified as an Objective Responder since the criterion is a 30% reduction. A continuous metric may provide a more meaningful interpretation of this data. **Figure 3E** shows a patient with prolonged stable disease. This patient likely benefits from therapy but is *not* considered a responder. **Figure 3F** shows a patient with transient progression that also appears to benefit from therapy but is also *not* considered an Objective Responder.

## Clinical data shows lesion-to-lesion heterogeneity

**Figure 4:**
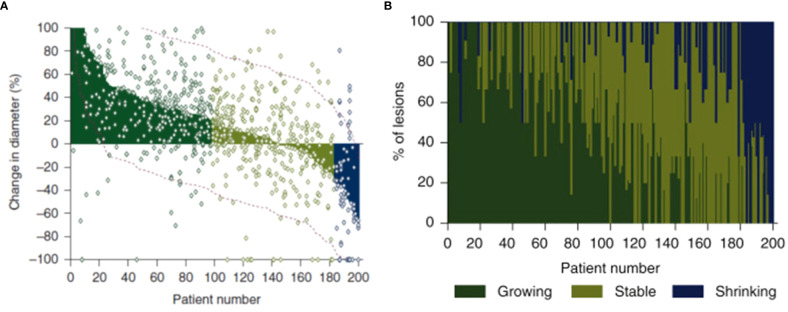
In these figures, patients are ordered from worst aggregate response (greatest dSLD, Patient#1) to the best aggregate response (least dSLD, Patient #200). In **(A)** we show the dSLD in solid coloring & the variability in individual lesion change in diameters is also show as dots. The red dotted lines represent standard deviation of the lesions change in per patient. In **(B)** we show the fraction of growing (change in diameter >20%), stable & shrinking (change in diameter < -20%) lesions in each of the patients. Reproduced (11) from with permission.

In a recent paper (11), we quantified lesion-to-lesion heterogeneity observed in patients with melanoma, NSCLC, and gastric cancer who were treated with pembrolizumab. Most patients displayed a mixture of growing, stable, and shrinking target lesions at the time of being classified as Progressive Disease (PD) (**Figure 4**). Figure shows a standard waterfall plot, with the bars representing the change in the sum of longest diameters for patients with melanoma that displayed primary progression (PD at first scan) on pembrolizumab therapy. Surprisingly, only 50% of these patients show a clinically meaningful increase in aggregate tumor burden (ΔSLD > +20%). More surprisingly, the change in size of individual lesions in these patients (dots) spanned a range of 120%. In other words, a patient with no net change in their sum of lesion diameters generally showed individual lesion responses ranging from -60% to +60%.

Other groups have also pointed out the limitations of RECIST for classifying heterogeneous responses to immunotherapy (sometimes calling it ‘dissociated response’) and called for better ways to evaluate patient response (12–16). Although new guidelines (like iRECIST) have been developed to improve and complement RECISTv1.1 in cancer immunotherapy trials, the lesion-to-lesion heterogeneity remains largely underappreciated.

## Implications of ignoring lesion-to-lesion heterogeneity in decision-making

### Misleading interpretations of biomarkers sampling

Biomarkers are used to identify responder populations and generate insight into the mechanisms driving success or failure. In IO trials, however, biomarkers are usually analyzed from an archived tumor sample or a non-target lesion sample (17–19). While biomarkers are occasionally evaluated in target lesions, they are generally analyzed at the patient level, rather than at the lesion level. For patients with lesion-to-lesion heterogeneity, it can be unclear whether the biomarker result was derived from a growing or shrinking lesion. In **Figure 2A**, every lesion is growing; the tumor sample is from a growing lesion. However, in **Figure 2B**, only half of the lesions are growing. It is unclear whether the tumor sample was derived from a growing or shrinking lesion. In **Figure 2C**, all lesions were shrinking; the tumor sample was derived from a shrinking lesion, despite the patient-level characterization of Progressive Disease (PD). Our analysis of lesion-to-lesion heterogeneity in melanoma shows that only ~50% of target lesions progressed in PD patients (11). This suggests that approximately 50% of biomarker results in RECIST PD patients may be from lesions that shrink on treatment.

Potential solutions to this problem include collecting biomarker samples from target lesions (so we know whether the sampled lesions grew or shrank and samples at the time of progression). However, given the challenges of lesion biopsies, measuring circulating biomarkers like ctDNA (20) at baseline and at the time of progression to identify changes in biomarker prevalence may be more practical. More work needs to be done to understand the relationship between these biomarkers and lesion heterogeneity.

### Does progressive disease justify drug discontinuation?

In early immune-oncology studies, RECISTv1.1 PD was listed as a cause for investigational treatment discontinuation that necessitated switching to a subsequent therapy. Several oncologists continued dosing despite the recommendation given that their patients were doing well, despite having Progressive Disease per RECISTv1.1 (21, 22). As a result, immunotherapy discontinuation decisions now rely upon alternative metrics, such as irRECIST, iRECIST, and WHO criteria (7, 23, 24).

Here, we propose a lesion-level treatment strategy. If all lesions are growing, the patient should switch to a new therapy. If all lesions are shrinking, the patient should remain on the existing therapy. Patients with a mixture of growing and shrinking lesions should do both (remain on existing while adding on a new therapy). and other such strategies for dealing with complexity and heterogeneity need to be tested in clinical trials as well as evaluated using in QSP models that account for lesion-to-lesion variability.

### Personalized medicine focused on addressing every lesion

We have argued (25), based on simulation analysis of lesion-to-lesion heterogeneity, that checkpoint combinations may be ineffective for patients whose immunologically ‘hot’ lesions are already shrinking due to pembrolizumab; non-responding ‘cold’ lesions may be unaffected by other immunotherapies that depend on T-cell activation. In contrast, immunotherapy combinations may be most impactful for patients with ‘intermediate’ T-cell infiltration (‘warm’ lesions) that are inadequately controlled by a single checkpoint. For other patients, therapies effective against cold tumors (e.g., chemotherapy, oncolytic viruses, targeted therapy) may be more effective combination agents to eliminate non-responding lesions. Understanding individual paths to progression is critical for determining the right combination therapy to offer to a patient. This may be facilitated by on-therapy biomarker samples from progressing lesions or ctDNA samples at the time of progression. Of note, decisions about continuing or switching treatment in the context of heterogeneous response across metastatic lesions should be made on a case-by-case basis, taking into consideration multiple factors such as the patient’s disease history, molecular profile, and treatment goals.

### Go/No-go decisions in drug development

Go/No-go decisions in early oncology drug development are driven by RECISTv1.1-based scores such as Objective Response Rate (ORR, % of patients who are classified CR+PR) and Progression-Free Survival (PFS) curves. Here we argue that patient-level (vs. lesion-level) definitions of efficacy can introduce bias into Go/No-Go decisions. First, RECISTv1.1 underestimates efficacy by classifying patients with mixed responses to therapy as having failed therapy. These patients benefit (in a subset of lesions), suggesting that the investigational agent may be effective as part of a combination therapy. Second, RECISTv1.1 underestimates efficacy in patients that are treated beyond progression. Approximately 50% of patients are treated beyond progression, many for prolonged periods of time (8). This suggests that many practitioners do not consider RECISTv1.1 PD as drug failure (lack or loss of efficacy). Third, ORR overestimates efficacy in patients who gain only transient benefit (due to rebound or intolerability). Chemotherapy, for example, tend to elicit better ORR but worse survival than ICI drugs. Finally, heterogeneity in biomarker samples can make it harder to identify responder populations.

We propose that the goal of cancer therapy is to shrink as many lesions as possible, as deeply as possible, for as long as possible. Thus, the first index in any improved scoring framework should include the percentage of lesions that are responding. It is important to know whether the investigational drug or combination controls a larger number of lesions per patient than standard of care regimens. The second and third indices are depth and duration of tumor response. This comprises an area under the curve calculation from the lesion-size-over-time spaghetti plot. However, instead of stopping the plot at the time of RECISTv1.1 progression, the curve would continue until the investigational drug is discontinued and a new therapy is initiated. This would provide high scores for patients who remain on therapy for prolonged periods of time, even RECISTv1.1 non-responders who display stable disease or transient progression.

## Framework to incorporate lesion-to-lesion heterogeneity in QSP models to support drug development

QSP models in Immune-Oncology have been used in various stages of drug development to support decision-making (26–29) They play a unique role in enabling integration of knowledge and data from multiple trials among the quantitative approaches available to drug developers. QSP model developers should consider incorporating the complex nature of clinical responses including lesion-to-lesion variability. This allows for simulated trajectories to capture the various paths to Progressive Disease (PD) seen in the clinical data and a more nuanced understanding of how a novel therapy has performed in a clinical trial.

We recently published two mathematical models of immune-mediated tumor killing that incorporate lesion-to-lesion heterogeneity. The first paper (25) investigated the limited utility of combination immune therapy in melanoma patients with a mixture of “hot” and “cold” lesions. The second paper evaluated the propensity for patients to benefit from treatment with pembrolizumab beyond progression based on the nature of their original progressive disease (30).

**Figure 5:**
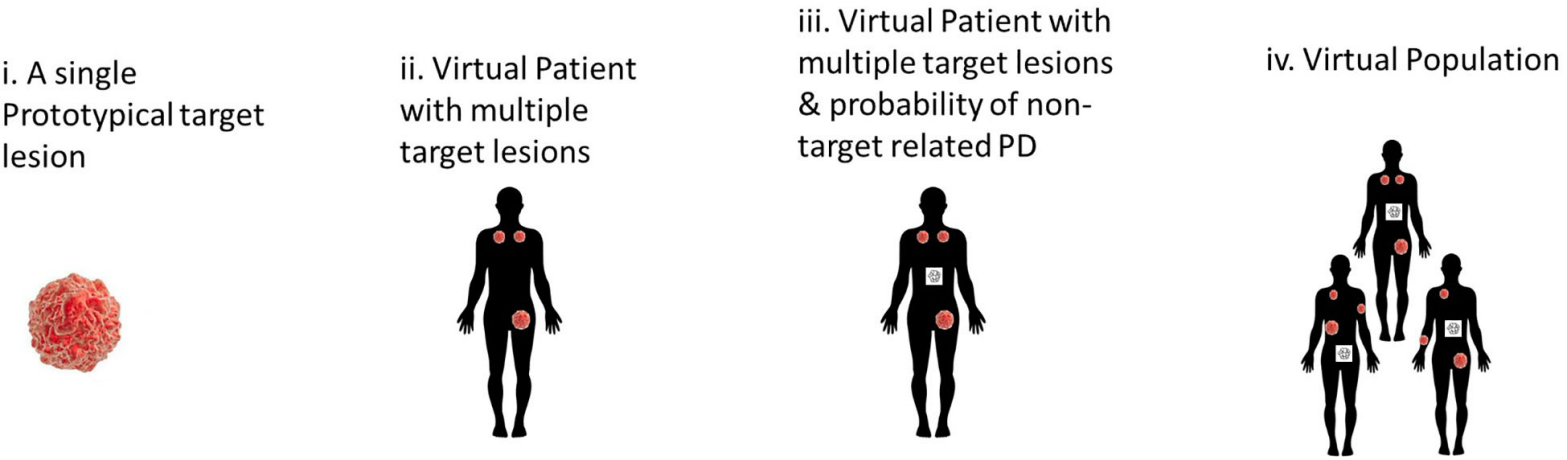
Hierarchical development of QSP model that provides a framework to incorporate lesion-to-lesion heterogeneity i. A single well-mixed lesion with interactions between tumor & immune system ii. Multiple target lesions within a single Virtual Patient tracked. The multiple target lesions have different growth rates, sizes etc. When ΔSLD>+20%, the patient is classified as PD iii. A stochastic model periodically predicts the probability of non-target driven PD (any one of non-target lesion growth or metastases or drop-out for other reasons). At this stage, the patient can be classified as PD when such an event occurs iv. A Virtual Population with such VPs that is calibrated to be consistent with reported clinical data – such as waterfall charts, RECIST scores, PFS curves.

The first paper developed a multi-scale model of tumor and immune cells interactions. In brief, the model included three levels: lesions, patients, and populations (**Figure 5**). This contrasts with models which have a single average lesion per patient. In each lesion, tumor cells were assumed to grow exponentially and can be killed by activated CD8 T cells. The multiple lesions within each patient were variable in many parameters, including initial number of cancer cells, growth rate, and immune infiltration (number of T Cells) (31). In addition, non-target lesion related factors leading to Progressive Disease were modeled as a probabilistic model dependent on tumor burden.

When QSP models are calibrated to capture population RECIST scores and progression over time, not explicitly accounting for individual causes of progression, it may hinder mechanistic understanding. For instance, a model of aggregate tumor burden may predict high efficacy for checkpoint combinations in most patients. However, when accounting for lesion-to-lesion variability, the model (25) predicted that adding ipilimumab to pembrolizumab had minimal effect on “hot” lesions (that responded well to pembrolizumab monotherapy) and cold lesions (that did not respond to either immune therapy). Instead, the combination was most effective in “warm” tumors and thus most impactful in patients with exclusively “hot” and “warm” tumors. These predictions need to be verified clinically but provide a framework to account for this complexity. In ongoing work, we connect such lesion level response to Progression Free Survival (PFS) and Overall Survival (OS) to gain additional mechanistic understanding and predictive capability (32).

The second paper evaluated salvage chemotherapy versus pembrolizumab beyond progression in a virtual clinical trial of patients with non-small cell lung cancer (NSCLC) who progressed on pembrolizumab (30). We applied empirical tumor growth models coupled with statistical sampling strategies to inform the probability of a given tumor lesion to respond to treatment beyond progression. Lesion level responses were simulated with organ-specific probabilities and magnitudes of response, as previously reported (14). Furthermore, a tumor-burden dependent probability of progression from non-target lesion growth or the appearance of new metastases was applied to facilitate the stratification of patients by the nature of their original progression.

**Figure 6:**
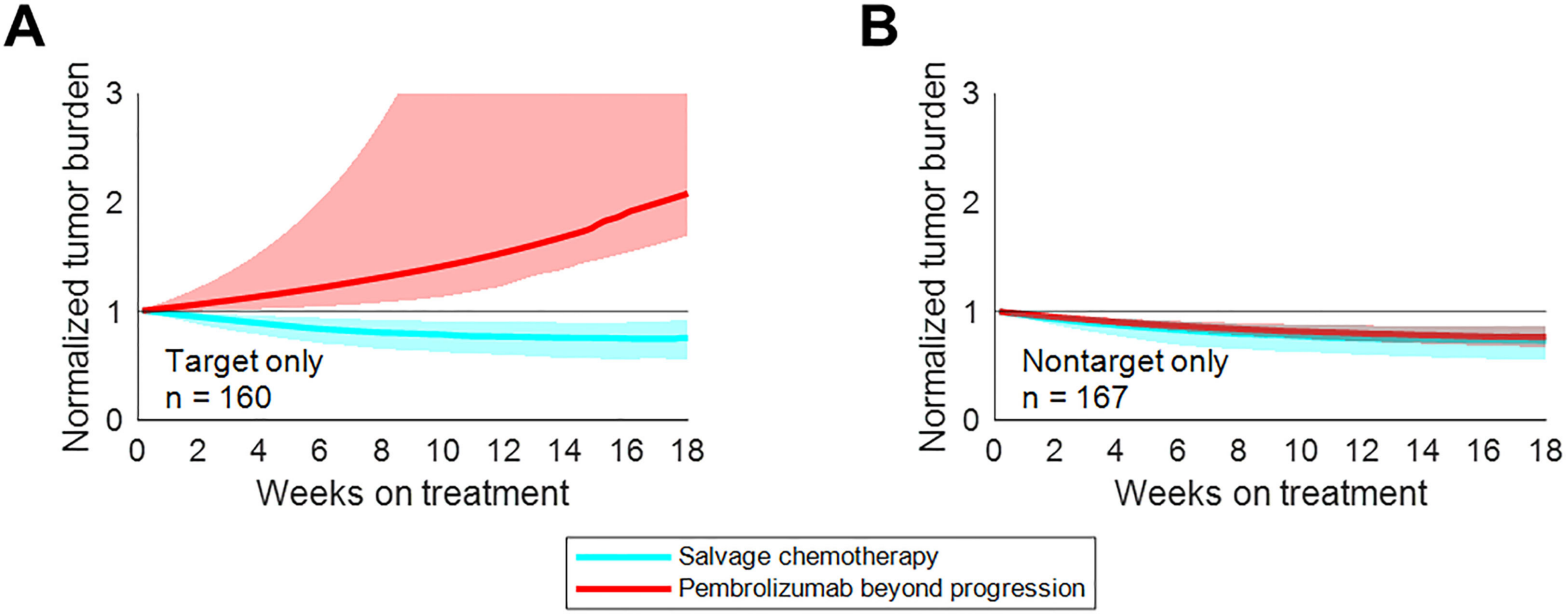
Treatment beyond progression may control tumor burden after nontarget progression on pembrolizumab. Median tumor burden in patients from an N=1000 simulated trial receiving pembrolizumab beyond progression (red) or salvage chemotherapy (cyan). **(A)** Patients with target progression without non-target progression or new metastases. **(B)** Patients with non-target progression or new metastases without target progression. Solid lines represent medians, while shaded regions indicate interquartile ranges. Reproduced from with permission.

While uniformly switching to salvage chemotherapy yielded better population-level outcomes than uniformly maintaining pembrolizumab beyond progression, there was a subset of patients for whom pembrolizumab beyond progression yielded longer progression-free survival. These patients tended to be those whose initial progression was due to non-target lesion growth or the appearance of new metastases – not those with target lesion growth (**Figure 6**). Prospective trials evaluating pembrolizumab beyond progression in this setting may be warranted.

Achieving systemic tumor control across all metastatic lesions is critical for long-term patient survival but remains a distant goal. High lesion-level response heterogeneity persists, conferring many dissociated responses across metastatic lesions. We developed a statistical metric - “Gower distance” to quantify response heterogeneity across metastatic lesion, which was found closely associated with drug efficacy and long-term patient survival (33). In addition, we developed mathematical models to investigate lesion-specific heterogeneity in terms of their dynamics in growth, response, and progression during treatment. We found that organ-level progression sequence is closely associated with long-term survival; in addition, patients with metastatic colorectal cancer whose first lesion-level progression occurs in the liver often have worse survival (15, 34).

Several groups have developed effective QSP models of immune-mediated tumor killing (35–38). Most QSP models develop Virtual Populations of patients with cancer and predict clinical outcomes assuming each patient has only a single average tumor (26, 27). This simplification may be appropriate in some cases (for example, in pre-clinical setting with a single lesion per animal or to explore tumor-immune interactions in a single lesion) but could result in misleading estimates of clinical efficacy when calibrated to RECISTv1.1 scores.

In the clinical setting, most patients display a mixed response due to inherent pathophysiological heterogeneity. Virtual patients in QSP models need to be more realistic to identify the combination therapies capable of generating broad and sustained responses in the clinic. It is encouraging to see more efforts in this direction (39).

## Summary and next steps

- Lesion-to-lesion heterogeneity remains underappreciated in oncology trials. Mounting evidence shows that RECISTv1.1 criteria are too broad to adequately characterize patient benefit from therapy, especially cancer immunotherapy. We should include individual lesion responses to improve the assessment of drug efficacy and patient benefit.
- Ignoring lesion-to-lesion heterogeneity could bias our decision-making process in oncology trials. We should interpret individual lesion-derived biomarkers with caution, as they may not reflect the characteristics of other lesions. More attention should be paid to patients categorized as progressors per RECISTv1.1, as many of their lesions may still be responding or have already stabilized. For these patients, combination therapy should be considered over discontinuation of current treatment.
- We should work together within our cancer research community to develop and validate more data-driven approaches to evaluate drug efficacy. Lesion-to-lesion heterogeneity should be considered during the QSP model development, clinical trial simulations, and statistical modeling to support better decision-making.

## Author contributions

All authors listed have made a substantial, direct, and intellectual contribution to the work, and approved it for publication.
